# Metallothioneins: Emerging Modulators in Immunity and Infection

**DOI:** 10.3390/ijms18102197

**Published:** 2017-10-23

**Authors:** Kavitha Subramanian Vignesh, George S. Deepe

**Affiliations:** Division of Infectious Diseases, College of Medicine, University of Cincinnati, Cincinnati, OH 45267, USA; george.deepe@uc.edu

**Keywords:** Metallothioneins, zinc, cytokines, signaling, infection, antimicrobial defenses, metals, nutritional immunity

## Abstract

Metallothioneins (MTs) are a family of metal-binding proteins virtually expressed in all organisms including prokaryotes, lower eukaryotes, invertebrates and mammals. These proteins regulate homeostasis of zinc (Zn) and copper (Cu), mitigate heavy metal poisoning, and alleviate superoxide stress. In recent years, MTs have emerged as an important, yet largely underappreciated, component of the immune system. Innate and adaptive immune cells regulate MTs in response to stress stimuli, cytokine signals and microbial challenge. Modulation of MTs in these cells in turn regulates metal ion release, transport and distribution, cellular redox status, enzyme function and cell signaling. While it is well established that the host strictly regulates availability of metal ions during microbial pathogenesis, we are only recently beginning to unravel the interplay between metal-regulatory pathways and immunological defenses. In this perspective, investigation of mechanisms that leverage the potential of MTs to orchestrate inflammatory responses and antimicrobial defenses has gained momentum. The purpose of this review, therefore, is to illumine the role of MTs in immune regulation. We discuss the mechanisms of MT induction and signaling in immune cells and explore the therapeutic potential of the MT-Zn axis in bolstering immune defenses against pathogens.

## 1. Introduction

Regulation of metal homeostasis is crucial in biological and cellular processes such as development and functions of organs, optimal enzyme activity, intracellular signaling and cell to cell communication [1]. Metallothioneins (MTs) are low molecular weight, cysteine-rich proteins that physiologically bind Zn and Cu in cells, but also sequester heavy metals such as cadmium (Cd) and mercury (Hg). They are induced by a variety of physiological and xenobiotic stimuli, buffer Zn and Cu ions, mitigate oxidative damage and protect against heavy metal intoxication [2]. In light of their ability to regulate metal ion availability, distribution and transport in cells, MTs have emerged as prominent players in maintaining overall organism fitness.

Development of mice genetically deficient in MT1/2 (MT-null) and MT3 isoforms has facilitated analysis of their functions in the resting state and in disease. In these models, in an unperturbed environment, MT deficiency does not result in apparent developmental, reproductive or age-related defects suggesting that MTs may be dispensable for normal development [3,4,5]. However, challenging MT-nullmice with common environmental stressors such as heavy metals, microbes or oxidative stress profoundly impacts fundamental processes such as DNA repair mechanisms, cell viability and inflammatory processes that rely on metal ion homeostasis and redox regulation for optimal functions [4,5,6,7]. For example, in mice and humans, Cd exposure induces MTs that sequester the metal to mitigate heavy metal poisoning. MT-null mice, however, exhibit decreased tolerance to Cd, and exposure results in nephrotoxicity and liver damage [3,4,8]. MT dysregulation is also observed under a wide spectrum of diseases including cancer, atherosclerosis, metabolic disease, autoimmunity, and infections [9,10,11,12,13,14]. Thus, it is clear that MTs respond to and are modulated in disease settings. However, a vast underlying gap in knowledge exists about the inducers and regulators of their complex functions in immunological responses. Deciphering how MTs shape the fate of development, dynamics and resolution of an immune response will be a crucial step in identifying novel therapeutic targets in pathways regulated by MTs. On the one hand, Zn and Cu have long been known to be involved in development and function of the innate and adaptive arms of our immune system [15] and on the other hand, numerous studies have reported MT regulation in the context of immunity [16]. However, our understanding of how this metalloprotein executes metal modulation in immune cells and molecular cues that drive these functions is fairly recent. The focus herein, stems from such recent insights into the fundamental role of mammalian MTs in immune regulation, with an emphasis on their ability to leverage host-pathogen interactions. We summarize the fundamental aspects of MT function and its role in Zn homeostasis (reviewed in greater detail elsewhere [2,17,18,19,20]) prior to exploring the interplay between MTs and immune responses.

## 2. The Metallothionein Family: Master Zinc Regulators

MTs are low molecular weight (6–7 kD), highly conserved, cysteine (Cys)-rich proteins that bind metals through thiol-clusters [17]. The MT protein family constitutes four isoforms (MT1–4) in mice and several isoforms with subtypes/variants in humans (MT1A, MT1B, MT1E, MT1F, MT1G1, MT1G2, MT1H, MT1HL1, MT1M, MT1X, MT2A, MT3, and MT4, and the pseudogenes *MT1DP*, *MT1JP*, *MT1L*, *MT2P1*, *MT1CP*, *MT1LP*, *MT1XP1*, *MT1P3*, *MT1P1* and *MTL3P*) [21,22,23,24].

In the early 1970s, a role for MTs in sequestering heavy metals such as Cd and Hg pinpointed their effect in alleviating xenobiotic stress [25]. Long since their discovery, the physiological functions of MTs remained unknown. Why did prokaryotes, lower eukaryotes and complex organisms evolve to express highly conserved thiol-rich proteins with an apparent crucial role in moderating heavy metal poisoning? The enigma was gradually dispelled when Zn homeostasis surfaced as being essential to all biological processes. About 10% of the mammalian genome encodes Zn binding proteins that regulate a diverse spectrum of biological functions [26]. Zn, unlike Cu and iron (Fe), is redox-inert, supporting evolutionary conservation of Zn binding sites in a large number of metalloproteins [27]. Undoubtedly, Zn availability is strictly regulated by MTs and Zn transporters [28,29]. MTs are master Zn regulators that sense intracellular cues and modulate Zn through sequestration, mobilization or release. The Zn binding constant of thionein was thought to be the highest (>3 × 10^13^/M) in biological systems, but, more recently, MTs have been shown to bind Zn ions sequentially with graded affinity and exist in metamorphic states [30,31]. The possibility of random occupancy of metal binding sites on MTs, followed by rearrangement of ions to a thermodynamically stable state has also been proposed [32]. Zn excess induces MTs, whereas Zn deficiency causes release of the metal from MTs, in effect scaling the intracellular Zn pool in response to cellular redox and energy state [33,34]. Intriguingly, Zn handling by MT1 and MT2 is distinct from that of MT3 [35]. The former sequesters Zn and readily releases only 1 Zn ion; the metal-thiolate cluster of MT3, however, assumes an “open conformation” to readily release Zn [35,36,37]. Moreover, Zn binding to MT3 is non-cooperative, suggesting that excess Zn may not stimulate saturation of all metal-ion binding sites in MT3 [38]. Thus, MT1/2 and MT3 share common ground in Zn regulation, but also exert discrete functions in scaling the intracellular Zn quota. The literature on MT4 function is scarce. Zn coordination by MT4 results in weaker folding of the protein compared to that of MT1; it has been suggested that MT4 may function as a Cu-thionein [39]. Of note, our knowledge of MT structure and function is gathered from an amalgamation of studies performed in solution, ex vivo, in vitro as well as in vivo. Thus, it is essential to recognize that the biology of MTs is highly complex; their behavior under different biochemical and cellular environments is likely heavily influenced by the nature of the stimulus, metal composition and redox environment.

Upstream of these events, the Zn-sensing metal-response element-binding transcription factor-1 (MTF-1) regulates MT expression to maintain precision in the size of the intracellular free Zn pool [40,41]. MTs mobilize Zn into the nucleus, mitochondria, Golgi apparatus, lysosomes, endoplasmic reticulum, cytosol and, possibly, zincosomes [42,43,44,45,46,47]. How MTs achieve this feat in intracellular compartments with diverse Zn demands is not clear. Their amino acid sequence lacks signals that dictate localization to specific organelles. The 3′ untranslated region of MT1 mRNA signals transcript localization to the perinuclear region, arming the ability of MT1 protein to gain entry into the nucleus [48]. Another mechanism explicating MT targeting is protein-protein interaction. While it is well established that peptides and small molecules such as glutathione, ATP and GTP interact with MT, other interacting partner proteins have also been identified [27,33,34]. MT3 interacts with proteins involved in heat shock response, secretion, signaling pathways, metabolic enzymes and chaperones [49]. Such associations enable MT targeting to the extracellular milieu. Indeed, MTs have been detected outside cells [50]; whether they are actively secreted or passively released as a result of compromised membrane integrity is unclear. Mounting evidence points to an active involvement of MTs in modulating extracellular cues [51,52,53]. Nonetheless, in the field of MT biology, several unknowns remain. Do cells export MTs in their apo-form or as MT-Zn/Cu complexes? How do cells sense the need to tune extracellular Zn availability? Stretching beyond metal-ion buffering, what functions do MTs execute extracellularly? The rapid response of MTs to changing redox potential and Zn demands justifies their presence in this environment. Outside cells, MTs may participate in regulating chemotaxis, signaling, cell–cell communication, and mitigating oxidative damage [50,54]. A detailed understanding of MT functions will open new arenas for exploring their therapeutic potential in a variety of inflammatory disease conditions including Alzheimer’s, coronary heart disease, arthritis, obesity, cancer and infections.

## 3. Immunity: Do Metallothioneins Take Center Stage?

Metal homeostasis, particularly Zn regulation, is essential for the development and adequate functioning of the innate and adaptive arms of immunity. The adverse impact of Zn deficiency on antibody production, cytokine production, chemotaxis, cell signaling, proliferation and functions of B, T helper (Th) and natural killer (NK) cells is well established [55,56,57,58,59]. Aberrant Zn regulation caused by Zn deficiency increases susceptibility to bacterial, viral and fungal infections, whereas Zn excess can exert toxic effects on immune cells [57,60]. MTs calibrate Zn availability; it is therefore conceivable that MTs are important regulators of immune cell function and promote immunological fitness. However, the underlying evidence supporting this hypothesis is still in its infancy.

Our current understanding of the role of MTs in immunity is fueled by studies on MT1 and MT2. In the mouse thymus, a primary lymphoid organ, MT expression peaks prior to thymic growth and wanes during thymic involution [61]. These changes parallel the impact of Zn on thymic mass, wherein Zn deficiency promotes thymic involution during aging [62]. The specific MT isoforms altered during thymic development and involution are undetermined, but the Zn-inducible nature of the MT response may result from dynamic changes in MT1 and MT2 expression [61,62]. The MT3 isoform, that is largely associated with neuronal functions, is also detected in the thymus [62]. However, MT3 is not Zn-inducible and data clearly elucidating the expression and functional roles of MT3 in the thymus are lacking. Overall, our understanding of how MTs and Zn control immunological functions in the thymus is limited and requires further investigation.

Thymic spatio-temporal regulation of MTs over the lifespan of an animal may impact T cell development and maturation in this organ. Thymic epithelial cells secrete thymulin, a Zn-dependent hormone [63]. MT expression in the thymic epithelial cells correlates with that of thymulin in humans and its expression is enhanced in thymomas [64,65]. Whether MTs regulate thymulin expression or vice versa is not known, but it may be postulated that in thymic epithelial cells, MTs deliver Zn to thymulin, whose function critically depends on the availability of this ion. Aberrant MT regulation may therefore impact downstream processes controlled by thymulin secretion such as T cell selection, differentiation and lymphocyte function. Interestingly, thymic abnormalities in MT-null mice have not been reported, albeit, this is a poorly studied field. An essential process in T cell selection is the presentation of self and non-self antigens by thymic non-lymphoid cells and professional antigen presenting cells (APCs) such as dendritic cells (DCs). Zinc influences the expression of major histocompatibility complex class (MHC)II on the surface of DCs [66,67]. A rise in intracellular Zn diminishes MHCII, while Zn chelation elevates it [66,67]. The occurrence of this phenomenon in DCs residing within the thymus remains unknown, but stimulates the proposition that MT-Zn sequestration may calibrate MHCII levels on DCs in the thymus, ultimately influencing thymic T cell selection.

The strategic placement of lymph nodes enables immunological surveillance and is the center for cross-talk between innate and adaptive immunity. Expression of different MT isoforms, their regulation and functional aspects in myeloid and lymphoid populations in the lymph nodes have not been characterized. Lymph-node associated MT expression is emerging as a prognostic marker in disease diagnosis, especially in patients with tumors. For example, MTs exhibit significant elevation in sentinel lymph node biopsies obtained from breast cancer and melanoma patients [68,69]. This modulation is prognostic and signals disease progression. It is noteworthy that the lymph node harbors a highly dynamic environment as circulating immune cells, signaling molecules and foreign agents continually drain in and out of these nodes through the lymphatic system. Thus, the myriad factors that condition the lymph node milieu may potentially influence MT regulation and function in macrophages, DCs, CD4^+^ Th cells, cytotoxic CD8^+^ cells and NK cells that enter and leave the lymph nodes. It may be conceived that MTs regulate Zn metabolism, proliferative, apoptotic, oxidative and nitrosative responses of these cells. However, as is evident, our understanding of MT regulation in lymph node biology is very limited and we may be far from uncovering the interplay between MT regulation and immune cell function in these tissues.

Only a handful of studies have shed light on MTs in the bone marrow, spleen and Peyer’s Patches; our knowledge of the functional significance of MTs in these organs is extremely scarce. Hematopoietic stem cells in the bone marrow produce progenitors that differentiate into cells of the myeloid and lymphoid compartments [70]. Although data directly linking MTs to the immunological functions of the bone marrow are lacking, several lines of evidence suggest that MT-Zn homeostasis may play a significant role: (i) Dietary Zn deficiency or chronic Zn exposure results in precursor-B and -T cell apoptosis in the bone marrow [71,72]. MT1 expression in the bone marrow of rats is modulated by dietary Zn status [73]. The absence of MTs may perturb bone marrow Zn homeostasis, unless compensatory Zn transporters and other metalloproteins replace the loss; (ii) Several Zn-dependent transcription factors dictate terminal differentiation of precursor cells in the bone marrow. Early growth response-1 (Egr-1), a Zn-dependent transcription factor promotes monocyte differentiation to macrophages [74,75]. In contrast, another Zn-finger transcription factor, growth factor independent-1 (Gfi-1), antagonizes monocyte/macrophage lineage commitment and promotes neutrophil differentiation [75]. A number of studies support a role for Zn transfer from MTs to other metalloproteins including Zn-dependent transcription factors [76,77,78]. As such, metalation of proteins residing in the Golgi and endoplasmic reticulum (ER) must occur in these organelles [46,79]. The nucleus is another site where Zn availability must be tightly regulated to mediate Zn binding/release by gene-inducer and repressor molecules and prevent oxidative DNA damage [80,81]. MTs are detected and/or regulate the functions of these organelles [42,43,82]. The finding that MT resides in the nucleus raises questions about its contribution to nuclear functions such as gene regulation and provides clues to its crucial influence in regulating the size of nuclear Zn pool. (iii) Mt-null mice contain fewer CD4^+^, CD8^+^ T and B cells in blood [83]. The ubiquitous expression of MT1 and MT2 and control of proliferative, apoptotic, oxidative and chemotactic responses imply that MTs potentially shape the bone marrow immunological milieu.

**Figure 1:**
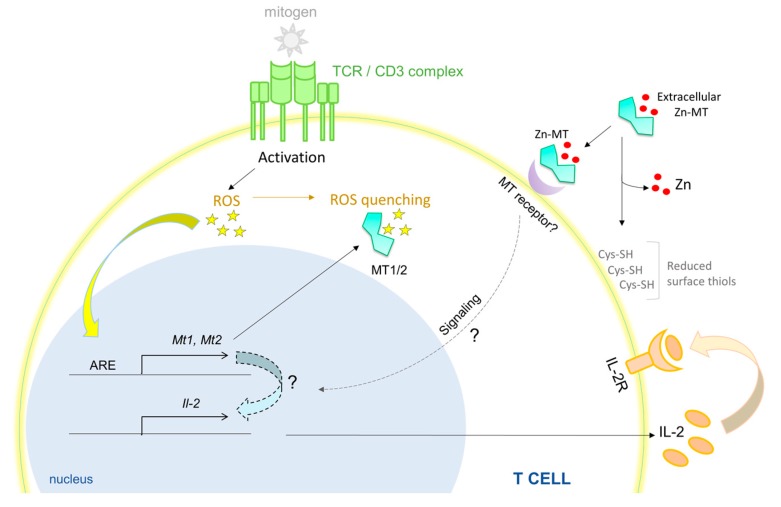
MTs (Metallothioneins) promote T cell survival and hyperproliferation. Activation of T cells with a T cell-specific mitogen generates ROS. MT1 and MT2 are induced by ROS produced during T cell activation, possibly by engagement of the antioxidant response elements (ARE) on the MT promoter. MTs quench ROS by oxidation of Cys residues, thereby preventing oxidative damage to T cells. MTs are required to produce optimal IL-2 that through autocrine and paracrine signaling, supports T cell survival. Exogenously added Zn-MT associates with the T cell membrane, possibly by binding to a yet unidentified MT receptor. Extracellular or membrane bound MTs reduce surface thiols (Cys-SH) on T cells. Mitigation of oxidative stress, reduction of surface thiols and an IL-2 response converge to promote T cell survival and hyper-proliferation by MTs. Red dots, Zn ions; all solid lines and arrows are known links; all dotted lines and arrows are predicted links.

The spleen is a major site for antibody production, erythrocyte clearance and filtration of pathogens. The splenic composition of CD4, CD8 and B cells in MT-null mice was found to be similar to that of WT animals under unperturbed conditions [84]. Contradictory findings have suggested that these MTs are involved in regulating splenic cellularity and function. One study reported elevated number of lymphoid cells and a 19% increase in splenic weight under MT1/2 deficiency. The number of circulating lymphocytes and splenic B cells were reduced in these mice, but number and proportion of splenic CD4^+^ and CD8^+^ T cells were marginally elevated [83]. Differences in age, gender and/or Zn concentration in the mouse diet used in the two studies may have led to discrepancies in the findings. Nonetheless, substantial evidence points to the role of MTs in regulating splenic T and B cell biology. MT-null splenic T cells fail to proliferate robustly upon stimulation with an antigen-independent T cell mitogen, concanavalin A or α-CD3. These splenocytes produce substantially lower interleukin (IL)-2 and the proliferative defect of purified T cells is reversed by addition of this cytokine [84]. Treatment of splenocytes with purified MT induces a hyper-proliferative response that is subdued in the presence of a reducing agent. Interestingly, while apo-MT, Zn-MT and Cd-MT comparably trigger proliferation, addition of Cu-MT inhibits this response [53], suggesting that MTs impart disparate effects pertinent to the metal bound to thionein. Accessibility to thiol groups on MTs is crucial in driving the lymphoproliferative response. Post activation, rapid T cell expansion is accompanied by superoxide burst [85]. The MT promoter possesses an antioxidant response element (ARE) that triggers MT expression in response to reactive oxygen species (ROS) [86]. An MT-ROS feedback loop may be operative in T cells, wherein basal MT levels mitigate oxidative damage, while ROS triggers the expression of MTs through ARE that feeds back to control the intracellular redox environment. Such a mechanism may operate to shield T cells from oxidative disruption of intracellular processes and improve T cell viability (Figure 1). Concentration, transport mechanisms and regulation of metals in the spleen await thorough investigation, but clearly, erythrocyte infiltration and hemoglobin metabolism in this organ place a demand on strictly regulating Fe homeostasis [87]. Although MT does not directly mobilize Fe, dietary Fe intake and MT1 expression share a reciprocal relationship in blood cells in rats, perhaps indicating changes in cellular Zn homeostasis or redox state [88,89]. Excess Fe interferes with Zn availability to proteins and vice versa, suggesting that, in addition to mechanisms that control Fe homeostasis, an MT-Zn axis may well be functional in sizing the Zn pool in this organ [90,91].

Contrary its role in promoting T cell proliferation, an immunosuppressive effect of MTs on humoral immune responses has been described. The first evidence of modulation of humoral immune responses by MTs came from in vivo studies on ovalbumin (OVA) injected mice treated with Zn-MT or Cd-MT. MT injection suppresses serum anti-OVA specific IgG responses [52]. Moreover, neutralization of MT1 and MT2 boosts IgG responses, indicating that MTs are a negative modulator of T-cell antigen-dependent humoral immunity. Complementing these data, MT-null mice mount a pronounced IgG and IgM humoral response to OVA injection, 58% greater than that observed in wild type mice [83]. Perhaps, the splenic environment of these mice increases the propensity of B cell differentiation to plasma cells. The precise mechanism(s) that escalate plasma cell differentiation is enigmatic. One postulate is that MTs regulate nuclear factor-kappa B (NF-κB) activation that hastens B cell differentiation into plasma cells. Although this hypothesis has not been specifically explored in B cells, splenocytes from naïve MT-null mice exhibit increased NF-κB p50 subunit activation. Additionally, stimulation of these splenocytes with phorbol myristate acetate (PMA) and a calcium (Ca) ionophore increases p50 and p65 subunit activation compared to wild type splenocytes [83].

How does the function of a metal regulatory protein intersect with the globally significant transcriptional regulator, NF-κB? At least two hypotheses may be projected: (i) Redox control by MTs calibrates NF-κB activation; and (ii) Intracellular Zn buffering by MTs modulates the NF-κB activation pathway. These postulates are centered on the premise that ROS produced by mitochondria and phagosomes have signaling functions that may be stimulatory or inhibitory in the NF-κB pathway. Mitochondrial ROS and NADPH oxidase-derived phagosomal ROS enhance IκB degradation in the cytoplasm, resulting in increased NF-κB activity [92]. Oxygen intermediates also inactivate protein tyrosine phosphatases and dual specificity phosphatases, in turn, sustaining kinase activation [92]. While these mechanisms prolong NF-κB activation, MTs could subdue this response by quenching ROS through oxidation of Cys residues and Zn release from MTs. Zn itself is redox inert, but release of the metal upon oxidation tunes the intracellular redox state [27,93]. The redox regulating capacity of MTs is approximately 50 times greater than that of the major antioxidant, glutathione (GSH) [94]. Thus, when MTs are lacking, elevated ROS likely signals activation of NF-κB and its downstream pathways—resulting in enhanced humoral responses. On the flip side, Zn import by Zn importer protein (ZIP)8 directly modulates NF-κB function by inhibiting IκB kinase (Iκκ) [95]. This mechanism results in increased IκB mediated degradation of NF-κB. Whether MTs contribute to this process by altering Zn availability is not known. One may predict that heightened labile Zn in immune cells lacking MTs subdues NF-κB signaling. In fibroblasts, MT deficiency results in reduced levels of p65 [96]. These studies suggest that the impact of MTs on NF-κB signaling may vary by cell type. Whatever be the case, how MTs manipulate signaling to dictate immunological fate clearly deserves attention.

Of note, primary and secondary lymphoid organs exhibit remarkable architectural organization that regulate the development and function of immune cells [97,98,99]. Determining MT distribution and localization in these organs may reveal how MT expression patterns interweave with the thymic, lymph node and splenic architecture, providing clues to the mechanisms by which MTs guide immune cell programming, metabolism, maturation and lineage commitment.

## 4. Metallothionein Induction and Signaling in Immunity

Immunological surveillance places a unique “rapid adaptation” demand on cells of the innate and adaptive immune system. For example, unlike a hepatocyte that resides in the hepatic tissue, circulating immune cells constantly traffic in and out of tissues, through the lymphatic system and into primary and secondary lymphoid organs [100,101]. This implies that, in addition to preparation for an immune response, these cells must adapt to an ever-changing extracellular biological milieu that varies in oxygen tension and concentrations of cytokines, chemokines, hormones, metabolites, other small molecules and metals. Zn concentrations can exhibit large variations between circulation and within tissues—as much as 9 μg/cc (9 μg/g) in whole-blood to 520 μg/g in prostrate tissue [101,102]. It is not surprising then, that extracellular cues and immune signals modulate MTs in response to stress and a rapidly changing Zn environment. Cytokines such as tumor necrosis factor (TNF)α, IL-1α, IL-6 and interferon (IFN)γ modulate MTs and Zn metabolism in non-immunological organs such as the hepatic tissue [103]. However, do these mediators signal changes in MTs within immune cells? An integrative analysis of all immunological modulators of MTs in immune cells is lacking, but mounting evidence indicates that cytokines have a profound impact on MT gene regulation and functions in both myeloid and lymphoid compartments [44,104,105].

In response to the pro-inflammatory or M1 cytokine, granulocyte macrophage colony stimulating factor (GM-CSF), macrophages upregulate MT1 and MT2, with the latter exhibiting heightened changes. While both MT1 and MT2 are ubiquitously expressed and respond to Zn, MT2 induction is pronounced in response to GM-CSF [44]. Why MT2 responds more strongly to cytokine stimulation is elusive, but one possible explanation is that basal MT1 expression in macrophages is already high, perhaps near saturation. Further studies are needed to pinpoint the specific roles of MT1 versus MT2 in tackling redox changes and Zn metabolism in macrophages. In MT-null peritoneal macrophages, lipopolysaccharide (LPS) fails to strongly induce TNFα, suggesting that MT1 and MT2 are required for a macrophage pro-inflammatory response [106]. Moreover, MT-null macrophages manifest gross defects in antigen-presentation, expression of MHCII and co-stimulatory CD80 and CD86 molecules and cytokine production [107].

**Figure 2:**
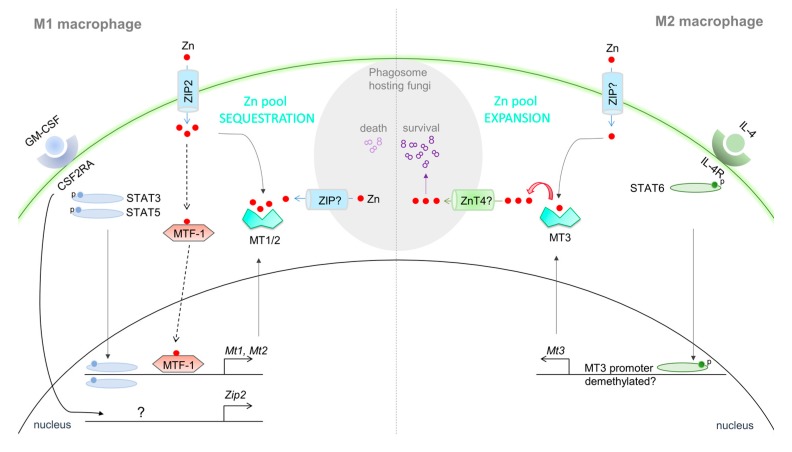
MTs shape the macrophage Zn pool and antifungal defenses. M1 macrophage (left side of center dotted line): GM-CSF binds to its receptor, CSF2RA, to trigger STAT3 and STAT5 activation. GM-CSF also upregulates Zn import via ZIP2. MT1 and MT2 induction is STAT3, STAT5 and ZIP2 dependent. While STAT3/5 can directly induce MTs by binding to the MT promoter, it is possible that ZIP2 mediated Zn import activates the transcription factor, MTF-1 to induce MT1 and MT2. In fungi infected macrophages, these MTs sequester Zn from *H. capsulatum* contained in phagosomes, possibly via Zn import into the cytosol, starving the pathogen of this essential element. M2 macrophage (right side of center dotted line): IL-4 binds to its receptor, IL-4RA to activate STAT6 mediated MT3 response. The *MT3* gene is not Zn inducible; IL-4 may prompt demethylation of the MT3 promoter to enable transcriptional activation by STAT6. M2 macrophages acquire Zn from extracellular sources that is rendered labile by MT3 and transported to the phagosome, possibly via ZnT4. This expanded labile Zn pool is exploited by *H. capsulatum* for survival within the macrophage. Red dots, Zn ions; all solid lines and arrows are known links; all dotted lines and arrows are predicted links.

Interestingly, the isoform of MT augmented in immune cells is pertinent to the nature of the stimulating signal. The anti-inflammatory M2 cytokine, IL-4, unlike GM-CSF, strongly induces MT3, but not MT1 and MT2 in macrophages [104]. The distinct Zn demand and defense functions of pro-inflammatory vs. anti-inflammatory macrophages may determine preferential expression of specific MTs. Indeed, MT1 and MT2 in M1 macrophages sequester the Zn pool, whereas MT3 in M2 macrophages serves to expand this fraction [44,104] (Figure 2). An immunological role for the MT4 isoform remains to be uncovered.

**Figure 3:**
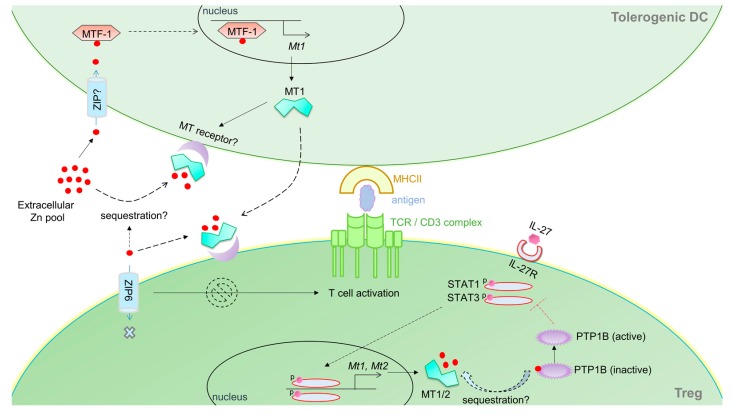
MT-Zn axis in tolerogenic DC–Treg interactions. DCs exposed to a pool of extracellular Zn develop a tolerogenic phenotype. Zn imported into DCs triggers MT-1 expression, possibly in an MTF-1 dependent manner. MT1 is expressed on the membrane of tolerogenic DCs and may also be actively secreted and bind to the T cell membrane. Upon activation, T cells import Zn via ZIP6 that mobilizes to the DC-T cell synapse. Zn sequestration by extracellular MTs may hinder this process, potentially stalling T cell activation. However, MT expression within T cells suppresses Treg development. IL-27 induces Tregs (in this case, IL-10 producing Tr1 cells) via STAT1 and STAT3 signaling. These STATs induce MT1 and MT2 that negatively feedback to inhibit STAT activation. MTs plausibly sequester Zn from PTP1B, an enzyme that is inhibited by Zn. Active PTP1B mediates dephosphorylation of STAT1 and STAT3 ultimately attenuating Treg development. Red dots, Zn ions; all solid lines and arrows are known links; all dotted lines and arrows are predicted links; red T bar depicts inhibition of STAT1/3 by PTP1B (active).

DCs bridge innate and adaptive immunity through professional antigen presentation and expression of costimulatory molecules. The many flavors of DCs execute very distinct functions, in effect, shaping the fate of adaptive T cell immunity [108]. For example, inflammatory DCs promote effector T cell responses, whereas tolerogenic DCs induce fork head box P3 (FoxP3) expressing regulatory T cells (Tregs) that suppress inflammation [109,110]. Mounting evidence suggests that DCs modulate MTs in response to molecular cues, and that they may, in fact, regulate their phenotype and function. LPS upregulates MT2A in mature and activated human DCs [111], but downregulates MT1 in mouse bone marrow derived DCs [112]. Murine DCs express MT1 in response to thermal stress, dexamethasone or ZnCl_2_ [112,113]. One may decipher that MT induction by stress modulates Zn distribution and regulates the intracellular redox environment in DCs. MTs (particularly MT1) promotes the development of tolerogenic DCs. These DCs express MT1 on their surface; blocking MT1 suppresses their tolerogenic potential, subduing naïve T cell differentiation into FoxP3 expressing Tregs [112]. This finding is intriguing because MT1 lacks a hydrophobic leader peptide sequence signaling surface expression or secretion. Whether DCs position MTs on the surface through interaction with other membrane proteins is not known. How MT expression on the DC surface confers a tolerogenic advantage also remains enigmatic. We and others have demonstrated that Zn handling in DCs is associated with modulation of DC phenotype [66,67]. DCs stimulated with LPS decrease their intracellular labile Zn pool leading to heightened surface MHCII expression. Overexpression of the Zn importer ZIP6 reverses this phenomenon [67]. Exposure of DCs to Zn salts also dampens surface MHCII, reduces pro-inflammatory cytokines such as IL-1β, IL-6 and IL-12 and promotes emergence of a tolerogenic signature characterized by increased programmed death ligand (PDL)1, PDL2 and enzyme indoleamine 2,3 dioxygenase expression [66]. Based on this premise, one may hypothesize that Zn, possibly via import through ZIP6, induces MTs in DCs that in turn regulate cellular processes associated with transport of MHCII containing vesicles. By localizing on the surface, MTs could gain access to the extracellular Zn pool, sequester it and subsequently interfere with Zn-dependent DC-T cell interactions. Indeed, superantigen presenting DCs rapidly trigger an ionic Zn signal that localizes to the subsynaptic region of the TCR activation complex in T cells [114]. Is it possible that MTs “soak up” Zn in extracellular spaces to stall TCR signaling? By scaling the size of the extracellular Zn pool, MTs potentially regulate T cell activation and proliferation in response to antigen presentation. Exogenously added MT binds to the membrane of purified CD4^+^ T cells [115]. Thus, it is also possible that DC-MT directly interacts with the T cell plasma membrane (Figure 3). In this case, MT may be envisioned as a co-stimulatory molecule on the surface of DCs. Perhaps, the protein reduces thiols on the surface of T cells that serve dual function: (i) mitigate oxidative damage to T cells; and (ii) calibrate ROS mediated T cell activation [115]. How such an interaction favors FoxP3^+^ Treg differentiation entails further investigation. Zn inhibits the histone deacetylase Sirt1, an enzyme that degrades FoxP3 in T cells [116]. Thus, on a paradoxical note, if MTs on the surface of DCs acted as Zn donors to T cells, the inhibitory effect of Zn on Sirt1 may in turn sustain FoxP3 expression.

MT expression within T cells regulates their activation, proliferation and differentiation potential. MT1 and MT2 manifest a late induction response in naïve T cells stimulated with IL-27 [105,117]. This cytokine stimulates generation of type-1 regulatory T (Tr)1 cells that drive immunosuppression via IL-10. In the absence of MT1 and MT2, Tr1 generation by IL-27 is greatly augmented, indicating that MTs thwart Tr1 differentiation [105]. In support of these data, exogenously added MT1/2 severely impair the emergence of IL-10 expressing Tr1 cells [118]. MTs interfere with signal transducer and activator of transcription (STAT)1 and STAT3 phosphorylation that are crucial for Tr1 generation [105]. The mechanism possibly involves modulation of Zn homeostasis by MTs. Numerous studies point to a role for Zn signaling in kinase and phosphatase functions [58,119]. In this view, one postulate is that by sequestering Zn, MT1 and MT2 sustain protein tyrosine phosphatase 1B (PTP1B) activity (an enzyme inhibited by Zn) that in turn dephosphorylates STATs (Figure 3). Indeed, MT-null Tr1 cells exhibit hyperphosphorylation of STAT1 and STAT3 and a pronounced IL-10 response [105]. T cells may orchestrate such a negative feedback mechanism through temporal control of MT expression. This idea is supported by at least two observations: (i) a late MT1 and MT2 response (past 48 h) is observed in Tr1 differentiating cells [105]; and (ii) early burst in the T cell–intracellular labile Zn pool is MT independent [117], and is likely governed by ZIP6 mediated Zn import. Contrary to its role in STAT inhibition, MT-driven Zn mobilization supports p38 MAPK signaling in Tr1 cells [117,120]. These data suggest that the MT-Zn pool plays distinct roles during early and late stages of Tr1 cell differentiation. It is noteworthy that MT1 and MT2 are also highly upregulated in the Th17 subset generated by IL-6 and TGFβ stimulation, but not in Th1 and Th2 cells [105]. In Th17 cells however, a clear role for MTs remains to be determined as MT-null Th17 cells develop normally and produce IL-17 [105]. Whether MTs dictate transcriptional programming, lineage commitment, regulate T cell plasticity and skew the Treg-Th17 balance remain open areas of investigation.

The immunomodulatory role of MTs is further highlighted by its impact on CD8^+^ T cell responses. Exposure of cytotoxic T lymphocytes (CTLs) to MTs diminishes surface MHCI and CD8 expression and impacts their ability to proliferate and mount cytotoxic responses against allogeneic target cells [121]. Zn deficiency reduces the proportion of CTLs in humans [122]. Moreover, in mice fed a Zn deficient diet, the ability of CTLs to kill tumor cells is impaired in vivo [123]. Taken together, our understanding of Zn-empowerment of CTL functions is vague, but the following postulates may be proposed: (i) MTs sequester Zn from the extracellular space, yielding a Zn-deficient environment; (ii) Zn deficiency alters membrane mobility, thereby affecting pore formation by perforins and exocytosis of cytolytic mediators by CTLs; and (iii) MTs quench ROS, in effect, impeding oxidative damage caused by CTLs. These postulates may well be interlinked, given the intimate relationship between MTs, Zn buffering and redox regulation. Although the data supporting MT mediated suppression of CTL functions are inclined towards an important role for Zn, the ability of MTs to donate Zn or modulate Cu homeostasis during CTL responses cannot be ruled out. MTs could additionally manipulate antigen presentation and interfere with surface receptors by formation of disulfide bridges with Cys residues, subsequently barring CTL-target cell interactions. Pending further research, these possibilities offer an opportunity to utilize MTs as therapeutic targets in disease conditions wherein CTL activity is crucial. For example, MT neutralization may benefit target cell killing by CTLs resulting in improved tumor outcomes or enhanced viral clearance.

Aside from its direct impact on immune cell behavior, MTs may act as chemoattractants to regulate cellular infiltration [54]. The arrangement of Cys residues in the MT molecule is reminiscent of its chemotactic properties; these motifs are also identified in chemokines. Clustal alignment of the MT protein sequence with that of C-C motif ligand (CCL)17 (both are encoded by genes on chromosome 8), reveals similarities in their Cys motifs, suggesting that MTs have a chemotactic attribute [54]. In vitro, exposure of Jurkat T cells to MTs results in F-actin reorganization and migration of the cells towards an MT gradient [54]. This phenomenon may be potentiated by direct MT and F-actin interactions [124]. In vivo evidence demonstrating MTs’ chemotactic potential is lacking, but, if true, why should the immune system utilize MT as a chemoattractant? This thought is intriguing, particularly because the immune system possesses a highly-organized array of chemokines and corresponding receptors that cater to cell infiltration demands. Perhaps, extracellular release of MTs is recognized as an “early alarm” by the immune system, a premise, supported by the observation that a variety of immunological stressors rapidly and strongly induce MTs. In light of their chemotactic potential, important questions emerge. How does extracellular MT communicate with immune cells? As indicated previously, MTs bind the surface of T and B cells [53]. Surface and nuclear receptors for MTs have been identified in astrocytes [125], however, there are no known receptors for MTs on immune cells. The chemotactic property of MTs can be blocked by cholera or pertussis toxins that also block G-protein coupled receptor signaling [54]. Thus, it is reasonable to propose that that MTs share chemokine binding sites on chemokine receptors. The specific chemokine receptors involved, the impact of metal occupancy on MTs on receptor binding and the downstream signals relayed by MTs in immune cells need to be dissected.

Thus, it is clear that MT regulation is intricately tied to immunological responsiveness. As discussed earlier, the classical inducer of MT1 and MT2 is the Zn responsive transcription factor, MTF-1 [41]. Nitric oxide (NO) produced by immune cells causes Zn release from MTs that activates MTF-1-dependent transcription of genes involved in Zn regulatory pathways, including MT1 and MT2 [126]. However, studies on MT regulation in MTF-1 deficient immune cells will be required to decipher whether MTF-1 is absolutely necessary for MT gene regulation. In addition to metal response elements (MRE), the MT promoter harbors sequences that respond to STATs, hypoxia, redox status and hormones, implying that immune cells are armored with additional means of MT regulation. TLR4 stimulation induces IL-6 dependent MT1 expression via STAT1 binding to the MT1 promoter [127]. Likewise, GM-CSF driven activation of STAT3 and STAT5 elevates MT1 and MT2 in macrophages. This response can be abrogated by RNA interference or pharmacological inhibition of STAT3 and STAT5 signaling. In these cells however, MT1 and MT2 expression is also governed by Zn import via ZIP2 [44]. STAT binding sites have been identified on ZIP genes [128]. Whether STAT3 and STAT5 drive ZIP2 expression in macrophages is not known. It may be proposed that these STATs provide the initial impetus to elevate *ZIP2* as well as *MT1* and *MT2* genes and Zn import by the former sustains expression of the latter via an MTF-1 dependent mechanism. In essence, there is active STAT3/5–Zn–MT crosstalk that shapes the “Zn sequestering” phenotype of GM-CSF activated M1 macrophages [44]. In M2-IL-4 polarized macrophages, STAT6 specifically elevates MT3, but not MT1 and MT2 expression [104]. Thus, through distinct signaling pathways, immune cells calibrate levels of different MT isoforms, in effect, shaping the intracellular Zn environment and redox status. Based on this premise, it is conceivable that STATs function as “Zn modulators”, whereby STAT signaling establishes the communication link between cytokines and Zn regulatory pathways in immune cells. Clearly, STAT activation plays a role in MT regulation (Figure 2), but the findings raise an intriguing question: Are MTs armored with the ability to modulate cytokine signaling? As discussed earlier, at least in the context of T cells, MT1 and MT2 subdue STAT1 and STAT3 activation during Tr1 differentiation [105] (Figure 3). The undisputed significance of Zn ions in enzyme function and cell signaling is reminiscent of the potential of MTs in controlling downstream effects of cytokine stimulation in immune cells.

Oxidative stress is intricately tied to innate and adaptive immunological functions. Rapidly dividing T cells heighten ROS production and phagocytes such as macrophages and neutrophils produce ROS to combat bacterial and fungal infections [85,129,130,131,132]. Thus, immune cells must mount antioxidant responses that selectively minimize damage to the host. Given its major antioxidant functions in addition to that of glutathione, MTs are rapidly induced by oxidative stress [93]. This response, is at least in part, driven by intracellular changes in Zn released from oxidized Cys and His residues on proteins that in turn modulate MTF-1 transcriptional activity. One contributor to this phenomenon is MT itself; the protein liberates Zn as a consequence of Cys oxidation [19,93]. Thus, MTs orchestrate a “self-amplifying response”, whereby mitigating oxidative stress activates a Zn-driven positive feedback loop to further propel MT synthesis. However, under oxidative stress, cells may not solely rely on Zn status to dictate MT expression. This proposition may be especially true in immune responses, wherein Zn changes promote key defense functions such as DC maturation and antimicrobial responses of macrophages and neutrophils [44,66,133,134]. The proximal promoters of *MT1* and *MT2* genes contain the ARE consensus sequence GTGACnnnGC. In the presence of H_2_O_2_, the basic helix-loop-helix-leucine zipper upstream stimulatory factor family (USF) protein recognizes a USF binding site that overlaps with ARE on the MT1 promoter to trigger transcription [135]. The roles that USF and ARE play in driving MTs during oxidative stress in immune cells is unknown. Perhaps, in an effort to avert oxidative disruption of crucial host immunological processes, transcriptional activation via both, MRE and ARE synergize to maximize MT production. Mouse *MT1* and *MT2* genes as well as the human *MT2* gene possess enhancer glucorticoid response elements (GREs) [136]. Glucocorticoids bind the glucocorticoid receptor (GR) to transcriptionally regulate genes involved in suppressing inflammation. HeLa cells respond to the synthetic glucocorticoid, dexamethasone, by inducing MT1 and MT2 that is further elevated by Zn supplementation [136]. In the context of immunity, these steroids have been widely used to treat autoimmunity and inflammation, primarily due to their ability to exert immunosuppression by influencing macrophage polarization, DC tolerogenicity, skewing Th1/Th2 responses and promoting IL-10 production by Tregs [137]. The significance of MTs in the suppressive impact of glucocorticoids remains unraveled. Do MTs promote or inhibit glucocorticoid mediated immunosuppression? Perhaps, the answer to this question lays in determining temporal—acute versus late phase—MT responses driven by GREs, MREs and AREs. The effect of dexamethasone parallels that of IL-27 and Vitamin D3 in differentiation of IL-10 generating Tr1 cells; these factors also promote MT1 and MT2 gene expression [105,117]. At least in the case of IL-27, MTs are late response genes and thwart emergence of suppressive immunity [105]. These data and ancillary evidence from studies on UVB radiation reveal that absence of MT1 and MT2 enhances immunosuppression [138], implying that MTs incline immune response progression towards “inflammation sustenance”. However, considering that MTs suppress B cell responses, the impact of MTs on humoral versus adaptive responses is discrete [83]. Deciphering the complex nature of MT regulation demands thorough investigation of how MT gene regulation, redox status, intracellular Zn levels intersect to influence signaling mechanisms and shape the outcome of inflammatory processes.

Evidently, a majority of the focus of MTs in immunity has rested on the functional significance of MT1 and MT2. This bias primarily stems from restricted association of MT3 with the CNS and a few other organs such as kidney and pancreas as opposed to ubiquitous MT1 and MT2 expression [139,140,141]; the former is also non-responsive to factors such as Zn and Cd that commonly induce MT1 and MT2. Epigenetic control has surfaced as another layer that regulates MTs; such a mechanism may especially be true for MT3 induction. This process has not been specifically investigated in different immune cells, but several lines of evidence point to a role for epigenetic machinery in influencing MT3 expression. Hypermethylation of CpG islands is observed upstream of the MT3 transcription start site in breast cancer epithelial cells, myeloid leukemic cells and esophageal adenocarcinomas and is associated with poor disease outcome [142,143,144]. Chromatin compaction in the DNA region where *MT3* gene resides possibly explicates the highly restricted nature of MT3 transcription. The finding that MT3 is a cytokine-inducible gene in macrophages suggests that polarizing stimuli manipulate the epigenetic structure of this gene, rendering it accessible to signaling mediators such as STAT6 [104,145]. MT1 may also be subject to such epigenetic control, whereby its induction is associated with extensive promoter demethylation [146]. Epigenetic regulation is a critical component of immune cell development and functions [147]. It is likely that immunological stimuli influence the epigenetic structure of MT promoters, adding another layer of complexity to the control of MTs during an immune response. However, the epigenetic status of MT promoters in different immune cell types and the impact of immune mediators on epigenetic control of MT expression is unknown. Nevertheless, a multitude of factors including the nature of the immunological stimulus, signaling environment, Zn availability and oxidative stress dictate the isoform of MT expressed and intracellular level of each isoform in immune cells. Notably, MT regulation in immune responses is frequently reported, but there exists a vast gap in our knowledge of the factors that regulate their expression and the functional significance of these proteins. For example, do immune cells employ multiple signaling platforms to modulate MT levels? How do immune cells distinguish the need to preferentially trigger MT1 and MT2 versus MT3 and what benefit does this confer? Are MTs involved in regulating fundamental differentiation and lineage commitment processes of macrophages, DCs and T cell subtypes? Finally, does MT4 play a role in immunity? Addressing these questions will necessitate analysis of MT functions using models that exhibit conditional deficiency of the MT isoforms in specific myeloid and lymphoid compartments. MT regulation is context dependent, is influenced by the stimulus in question and controlled at multiple levels of gene regulation, translation and protein turnover. It is crucial to factor these in, especially when data about MT functions are extrapolated from one immunological setting to another.

## 5. Metallothioneins Respond to Microbial Stress

A primary function of immune surveillance is to screen microbial invaders and rapidly respond by activating myriad immune pathways that converge to mount effective antimicrobial defenses. Microbial challenge is thus, a potent “stressor” that immune cells must be prepared to cope with or alleviate to maintain the hosts’ integrity during host-pathogen interactions. On the innate end, nutritional immunity and ROS are two principal defense mechanisms deployed by immune cells to curtail microbial growth and dissemination [132,148,149]. It is not surprising then, that numerous studies have reported dramatic upregulation of MTs, particularly MT1 and MT2 isoforms during bacterial, viral and fungal infections [44,103,150,151]. A classic example is hepatic MT1 and MT2 elevation during endotoxin (LPS) challenge. This is an acute phase response associated with rapid reduction in plasma Zn and ~29% increase in hepatic Zn. Production of IL-1α, IL-1β, TNFα and IL-6 precede this response and each of these cytokines independently trigger LPS driven MT transcription [96]. Additionally, in children with sepsis, MT elevation is a predictor of survival [152]. While precise pathways that link the cytokine response to MT induction remain elusive, hyperglucagonemia is a feature of endotoxin challenge that regulates MTs. In this view, a role for metabolic alterations and elevated blood glucose in modulating MT levels upon endotoxin challenge may be proposed. Indeed, several immune mediators impose unique metabolic demands on glucose and fat utilization to cope with inflammatory stress [153,154]. Another notable acute phase response during endotoxemia is the rapid elevation in ZIP14 that imports Zn into hepatocytes [155]. MT elevation likely signifies sequestration and retention of the newly imported Zn, as mice lacking MT1 and MT2 exhibit hepatic Zn loss and concomitant rise in plasma Zn [103]. This peculiar phenomenon confers, at least, dual benefit to the host: first, it restricts Zn access to pathogens in the extracellular environment; and, second, Zn sequestration potentially favors chemotactic migration of immune cells towards the site of inflammation. The former surfaces as an arm of nutritional immunity, wherein the immune system attempts to starve microbial invaders by withholding nutrients essential to biological processes. Localized Zn redistribution by MTs may shape the course of immune response by escalating DC maturation and skewing T cell responses towards that of an inflammatory phenotype. MT-dependent hypozincemia has similarly been reported in other gram-negative infection models including *Salmonella typhimurium* and the live vaccine strain of *Francisella tularensis* [156], implying a broadly applicable role of MTs in orchestrating hypozincemic responses upon bacterial challenge. The MT response is transient; withdrawal of the endotoxin stimulus results in rapid degradation of accumulated MT justifying the short half-life of Zn-thionein complex [154]. The fate of liberated Zn, however, is obscure. One obvious consequence appears to be redistribution and restoration of Zn homeostasis after infection has subsided. Beyond these, is there a role for Zn in suppressing pro-inflammatory responses and promoting tissue repair? If yes, do MTs partake in this process? Zn has a well-recognized role in wound healing and inflammation. Experimental models of Zn deficiency and Zn deficient individuals exhibit markedly delayed tissue repair [157,158]. Moreover, recent revelations of the role of MTs in wound-healing M2 macrophages, discussed in the following paragraphs, illuminate this possibility [104].

Perhaps, the impact of MTs on immune responses to infection is highly complex, depending on the cell type, regulation dynamics, degree of induction and the pathogen in question. On the one hand, in mice infected with the gram-negative bacterium *Helicobacter pylori*, MT1 and MT2 do not contribute to bacterial clearance but confer protection against erosive lesions in the gastric epithelium [159]. In this model, a lack of MTs aggravates infiltration of inflammatory cells, enhances macrophage inflammatory protein (MIP)-1α and monocyte chemoattractant protein (MCP)-1 expression and augments NF-κB DNA binding activity in the stomach tissue. How Zn buffering and antioxidant mechanisms of MTs converge to modulate the inflammatory process to yield subdued ulceration awaits further experimentation. On the other hand, genetic deficiency of MT1 and MT2 or increased MT gene dose, accelerate clearance of the gram-positive bacterium, *Listeria monocytogenes* in mice [160]. Thus, swaying MT expression in either direction favorably promotes antibacterial defenses. This observation may be explicated by disrupted redox control, as prolonged MT deficiency or MT overdose interferes with alleviation of oxidative stress. Indeed, the splenic lymphocyte population exhibits increased oxidized surface thiols in MT-null mice and augmented ROS in MT overexpressing mice [160].

Complementing the aforementioned studies on bacteria, a plethora of studies have revealed MT modulation in viral infections. Influenza A/PR8 strain that causes upper respiratory tract and lung infections strongly elevates MT1 and MT2 in the lung and liver [86]. Infection triggers engagement of MREs, ARE, GRE and STAT3 binding sites on the MT promoters, perhaps, signifying an effort to rapidly maximize MT production to curtail oxidative injury caused by heightened inflammation [86]. This hypothesis is further supported by an inverse correlation between hepatic MT expression and hepatitis C virus (HCV) pathogenesis and that MTs improve response of chronic HCV patients to IFNα therapy [161,162]. In an experimental model of human coxsackievirus B type 3 infection, MT1 and MT2 are augmented in the liver, kidney and spleens of mice, facilitating Cu and Zn redistribution across different organs [163]. Metal ion redistribution is frequently reported but how such a process benefits the host is enigmatic [164]. Viruses must explicitly supply all of their metal ion demands through the host metal pool; thus, it is reasonable to predict that changes in MTs and Zn or Cu redistribution are “virus-induced” phenomena. However, from the hosts’ perspective, can such a mechanism deter viral binding and dissemination? Studies on respiratory syncytial virus reveal an inhibitory role for Zn but not Ca, magnesium (Mg) and manganese (Mn) in viral replication [165]. Zn-ejecting compounds inhibit the activity of the transcription anti-termination cofactor protein, M2-1 that is crucial for viral RNA-dependent RNA polymerase function [166]. Zn also directly inhibits binding of common cold causing rhinoviruses to intracellular adhesion molecule-1 (ICAM1) receptor in the nasal epithelium, stalling viral entry into the host [167]. However, to directly obstruct viral entry, MTs must be present extracellularly. Whether cells release small amounts of MTs extracellularly at barrier interfaces in preparation for antiviral responses is unknown. Upon sensing viral insult, it is possible that some of the upregulated MT protein is released from cells to curtail further viral invasion. Nonetheless, a number of questions will need to be addressed. What signals MT release and how does the host decide how much should be released during infection? What is the fate of extracellular MTs after infection has subsided? Do viruses encapsulate host MTs as they are released from cells? The latter, if true, may be exploited as a mechanism of piracy by viral invaders that depletes the host of intracellular MT stores and manipulates MT levels in the extracellular space. An increased focus on Zn binding proteins and Zn ionophores in the development of antivirals suggests that harnessing MTs and their Zn modulating potential may prove beneficial to the host [168,169]. However, a thorough understanding of how MTs manipulate the course of an antiviral response will be necessary to pinpoint precise molecular processes involved in MT-mediated host-protection.

Immune responses against microbial challenge modulates host-MTs, but very little is known about the contribution and functions of immune-cell derived MTs in this context. The pulmonary fungal pathogen *Histoplasma capsulatum* establishes a replicative niche within macrophages prior to pro-inflammatory activation by cytokines such as IFNγ or GM-CSF [44,170]. The latter purposes as a “Zn reprogramming signal” that induces MT1 and MT2 to deplete intracellular labile Zn that would otherwise be readily accessible to *H. capsulatum* containing phagosomes (Figure 2). As indicated earlier, GM-CSF also triggers Zn import via ZIP2 in macrophages. This finding appears paradoxical, but the Zn flux probably serves to meet an increased demand of Zn-dependent processes that arm the macrophage defense machinery. In the host-pathogen combat, the Zn pool could be envisioned as “drifting” towards the former; one apparent advantage of this mechanism is that the host is guarded against oxidative disruption of critical cellular processes, while rendering the pathogen in a Zn-starved state [171]. A macrophage superoxide-boost is interwoven into the umbrella of MT-driven effects that ultimately cripples fungal replication [44,172]. Parallel to this phenomenon, is the finding that MT1 and MT2 detain intracellular labile Zn from *S. typhimurium* within macrophages [173]. Genetic deficiency of MT1 and MT2 elevates labile Zn and impairs oxidative and nitrosative macrophage defenses, a situation, that *S. typhimurium* exploits to acquire Zn and colonize the macrophage. Even in a Zn-restricted state, *S. typhimurium* secures the metal ion via the ZnuABC Zn importer [174]. On the flip side, macrophages also employ “Zn stress” to poison invading microbes. Human macrophages hosting *Mycobacterium tuberculosis* rapidly deploy Zn into the phagosome to intoxicate the pathogen. A similar mechanism is functional in *E. coli* eradication by macrophages [150]. Contrary to ZIP2 mediated Zn import in *H. capsulatum* infected macrophages, the source of this Zn pool is intracellular [44,150]. In these cells, MTF-1 localizes to the nucleus and various MT transcripts (*MT1H*, *MT1M*, *MT1X*, and *MT2A*) are elevated. However, whether MTs contribute to the Zn-burst is unclear. It has been proposed that ROS liberates Zn from MT-Zn complexes; the metal ion is channeled into the phagosome by yet unknown transport mechanism(s) to drive mycobacterial poisoning [150]. It is conceivable that cytosolic Zn is redirected by means of Zn transporters, but what signals the entry of this ion into vacuoles housing mycobacteria? Do MTs localize within late-endosomes and lysosomes, or do they bind to the phagosomal membrane and interact with transporters to guide entry of Zn into these vacuoles? Are extracellular Zn-MT complexes and mycobacteria “co-engulfed” as a result of phagocytosis? Regardless of the mechanism in action, it is reasonable to postulate that MT1 and MT2 arm phagocyte defenses, particularly that of macrophages. Pertinent to the pathogen in context, the precise molecular cues that instruct Zn poisoning as opposed to Zn sequestration by MTs remain enigmatic, but likely represents a carefully elected immunological decision that caters to incline Zn concentrations beyond the narrow “tolerance” window that succumbs pathogens to Zn-starvation versus Zn-overload (Figure 2). 

From the pathogens’ standpoint, survival within the host demands subversion strategies. In the case of mycobacteria, alleviation of Zn poisoning is achieved by activation of P1-type ATPases [150]. Interestingly, contrary to the hosts’ Zn-restriction defense strategy reported for *S. typhimurium*, macrophages also attempt a Zn-overload defense mechanism to eliminate this pathogen [175]. The involvement of MTs in this situation has not been deciphered, but the pathogen evades this response by virtue of Salmonella pathogenicity islands, expression of the bacterial Zn exporter, ZntA and yet unidentified mechanisms [175]. Thus, in their struggle for survival, pathogens launch counter-strategies that rely on Zn regulation to subvert host defenses. Given the highly conserved nature of MTs from single-celled to multicellular organisms, that pathogen-derived MTs are determinants of virulence within the host is an interesting conjecture to be explored. The finding that the fungal pathogen, *Cryptococcus neoformans* produces Cu-binding MTs to neutralize toxic copper-overload imposed by host macrophages is a classic premise demonstrating that MTs may also belong to the pathogen-defense armor [176].

Over half a century of research has emphasized the roles of MT1 and MT2 in immune responses. Until recently, virtually nothing was known about the contribution of MT3 in host defenses. Existing data on the regulatory and functional attributes of MT3 suggests that from an immunity point of view, the impact of MT1 and MT2 versus MT3 on host immunity is unparalleled. In M2 macrophages polarized with IL-4 or IL-13, MT3 is elevated and enriches the labile-Zn pool [104]. These type-2 cytokines promote parasite clearance, wound healing and tissue repair, but exaggerated IL-4 and IL-13 production worsen allergic inflammation and render the host susceptible to invasion by intracellular pathogens [177,178]. M2 macrophages harbor a favorable niche for the survival of *H. capsulatum*. Although the regulation of transferrin and arginase in these cells imply roles for iron metabolism and subdued NO defenses [179,180], emerging evidence pinpoints to the significance of Zn homeostasis by MT3 in suppressing macrophage defenses. M2 macrophages source the metal ion from the extracellular environment, but instead of sequestering it, render it readily accessible to the pathogen. This “expansion” effect on the Zn-pool is MT3-dependent and is at least partially driven by cathepsins that trigger Zn release from the protein. The pathogen ultimately gains access to the host-Zn resource, possibly via the transporter ZnT4, to thrive within phagosomes of M2 macrophages [104]. MT1 and MT2 act in stark contrast in M1 macrophages invaded by *H. capsulatum*; in these cells, Zn that once belonged to the pathogen befits the macrophage Zn-pool [44,104] (Figure 2). It is intriguing that, rather than being poisoned, this fungal pathogen exploits Zn-release tactics of MT3 to its advantage. The findings seed a fundamental query: Why did M2 phagocytes evolve to increase the labile-Zn pool? The existence of such a Zn modulatory machinery in M2 macrophages is reminiscent of the importance of Zn in anti-parasitic defenses [181]. Nonetheless, much remains to be unraveled about the involvement of the MT3-Zn axis in immune processes regulated by IL-4 and IL-13 signaling.

## 6. Concluding Remarks

The evolutionary web of MTs has casted its presence on virtually every life form—prokaryotes to lower eukaryotes, invertebrates to higher vertebrates including mammals. Within its lifespan, a living cell must adequately tap the potential of highly dynamic redox chemistries and metal ion environments to conduct various biological processes. Evidently then, through duplication and functional segregation, a number of MT isoforms emerged to support metal-ion buffering, maintaining homeostasis and protecting the host from oxidative assault. The complexity of MT evolution is readily demonstrated by differences in the MT gene clusters, copy number and functional characteristics between different species within the same genera [182]. The mouse has four MT isoforms, but numerous isoforms and subtypes/variants contribute to the heterogeneity of this family in humans; that newer proteins may line the MT queue pending annotation and functional characterization may not be a surprise. The question is: Will the MT family evolve further, and why? From a mammalian-immunity viewpoint, innate and adaptive barriers are constantly confronted with tremendous evolutionary pressure from environmental cues such as xenobionts including pathogens that rapidly manipulate their genomes and virulence strategies to sustain infection [183]. While direct evidence that immunological pressure imposed divergence of the MT family remains to be discovered, the >10 trillion microbes that have harmlessly populated the human gut and skin for eons suggests that such an evolutionary demand may have aroused within the host itself. Indeed, MT expression in the gut, known to be guided largely by dietary Zn status, maintains integrity of the gut epithelium, an important barrier that guards against dissemination of opportunistic pathogens [184,185]. On a captivating contrary note, is it possible that, evolutionarily, MTs exerted selective pressure to shape the human microbiome? The fundamental differences in metal-ion requirements, susceptibility to sequestration or intoxication and ability to withstand superoxide damage between microbes provide a framework to test this hypothesis. Nonetheless, investigating this possibility demands a rigorous understanding of microbial metal-ion homeostasis and how immune responses employ MTs to lay “beneficial” microflora within the host. Perhaps, the functions of MTs are intricately interwoven into the complex attributes of innate and adaptive cells; thus, in the world of immunity, we may have only scraped the surface of vast perturbations that underlie a dysregulated MT response. With careful consideration to how MTs globally impact the immune response, unearthing the potential of MT as an “antimicrobial protein” will take the next leap forward in battling the rising menace of antibiotic resistance and emerging virulent pathogens. Such a proposition must be supported by an unparalleled understanding of interactions of MTs with microbes and immune cells, half-life, fate of the protein and significance of its apo- and metal-bound forms in bolstering immunological defenses. Finally, gaining deeper insight into the immunoregulatory role of MTs holds promise in levitating this class of proteins in the therapeutic ladder targeting infections and the myriad diseases that engage host immunity.

## Abbreviations

APC	antigen presenting cell
ARE	antioxidant response element
Ca	calcium
CCL	C-C motif ligand
Cd	cadmium
CTL	cytotoxic T lymphocyte
Cu	copper
Cys	cysteine
DC	dendritic cell
Egr-1	early growth response-1
ER	endoplasmic reticulum
Fe	iron
FoxP3	Fork head box P3
Gfi-1	growth factor independent-1
GM-CSF	granulocyte macrophage-colony stimulating factor
GRE	glucocorticoid response element
GSH	glutathione (reduced)
HCV	hepatitis C virus
Hg	mercury
ICAM-1	intracellular adhesion molecule-1
IFN	interferon
IL-	interleukin
IκB	inhibitor of κB
Iκκ	IκB kinase
LPS	lipopolysaccharide
MCP	monocyte chemotactic protein
Mg	magnesium
MHC	major histocompatibility class
MIP	macrophage inflammatory protein
Mn	manganese
MRE	metal response element
MT	Metallothionein
MT-null	MT1/2 deficient
MTF-1	metal-response element-binding transcription factor-1
NF-κB	nuclear factor-kappa B
NK	natural killer
NO	nitric oxide
PMA	phorbol myristate acetate
PTP	protein tyrosine phosphatase
ROS	reactive oxygen species
STAT	signal transducer and activator of transcription
Th	T helper cell
TNF	tumor necrosis factor,
Tr1	Type-1 regulatory T cell
Treg	regulatory T cell
USF	upstream stimulatory factor
ZIP	zinc importer
Zn	zinc
